# Who leads exercise for older adults? A comparative policy and workforce analysis of older adult exercise leadership in Hong Kong, Taiwan, Japan, and Singapore

**DOI:** 10.3389/fpubh.2026.1879234

**Published:** 2026-08-07

**Authors:** Yi Chen Chiu, Ya Chun Shen

**Affiliations:** Department of Leisure and Recreation Management, Taipei City University of Science and Technology, Taipei, Taiwan

**Keywords:** comparative institutional analysis, cross-sector referral, exercise leadership, older adult exercise services, professional roles, workforce governance

## Abstract

**Background:**

As population aging accelerates across Asia, exercise services for older adults must address not only participation but also which professionals should provide support for individuals with different health and functional needs.

**Methods:**

This study used an integrative review and comparative institutional analysis to examine workforce arrangements for older adult exercise services in Hong Kong, Taiwan, Japan, and Singapore. Twenty-three academic and gray-literature sources were identified through a PRISMA-ScR-informed process and appraised using AACODS and CASP-informed criteria.

**Results:**

Hong Kong mainly reflected a community-based fitness-extension model, Taiwan linked fitness provision with disability and long-term care prevention, Japan demonstrated the strongest documented connection between specialized health-exercise roles and collaboration with health professionals, and Singapore emphasized public health promotion and coaching development. Across all four jurisdictions, service pathways were clearer for relatively healthy and independent older adults than for those with frailty, multimorbidity, recurrent falls, functional decline, or other complex needs. Risk stratification, scope of practice, referral, monitoring, and shared-care arrangements were inconsistently defined.

**Discussion:**

The study proposes a tiered service and competency framework that matches provider expertise with participant risk. General fitness and community personnel may support lower-risk older adults, whereas individuals with more complex needs require advanced exercise expertise or interprofessional management. More explicit allocation of assessment, monitoring, escalation, referral, and collaborative responsibilities is needed to support safe and sustainable exercise services.

## 1 Introduction

As population aging accelerates across Asia, healthy aging has become both a demographic concern and a governance challenge involving health, care, social participation, and institutional adaptation. International policy has consequently shifted from extending life expectancy alone toward maintaining functional ability, delaying disability, and supporting independent living in later life (1). Physical activity is central to this agenda because of its contributions to chronic disease prevention, mental health, mobility, balance, functional maintenance, and quality of life (1, 2).

Physical activity and exercise nevertheless involve different forms of provision. Physical activity encompasses bodily movement undertaken in daily living, transport, recreation, work, or leisure, whereas exercise is planned, structured, repetitive, and undertaken to maintain or improve fitness or health. Exercise prescription additionally specifies the type, frequency, intensity, duration, and goals of exercise, together with appropriate monitoring and follow-up (3). Population-level recommendations may therefore be implemented through self-directed activity and public health promotion, while structured exercise programmes may require assessment, programme design, progression, modification, monitoring, and professional supervision. The workforce required depends on the purpose, setting, complexity, and risk level of the service.

This distinction is particularly important because older adults vary widely in health and functional status. Many are medically stable and functionally independent and can participate in community fitness, recreational exercise, or health-promotion programmes with limited supervision. Others live with frailty, sarcopenia, impaired balance, recurrent falls, multimorbidity, functional limitations, medication-related concerns, or recent medical treatment (1, 4–6). These conditions can affect exercise tolerance, contraindications, progression, monitoring needs, and decisions about referral. Providers may therefore require different levels of competence in health-history review, functional assessment, individualized prescription, symptom monitoring, exercise modification, and communication with healthcare professionals (7).

International clinical exercise models illustrate how such responsibilities can be differentiated. In Australia, general exercise professionals commonly serve apparently healthy or medically stable individuals, whereas Accredited Exercise Physiologists undertake university-based education, supervised clinical training, and professional accreditation to provide exercise interventions for people with chronic, complex, or higher-risk conditions (8–10). This distinction does not imply that all older adults require clinical supervision. It demonstrates how workforce arrangements can match professional preparation with participant need and create clearer boundaries between general exercise provision and services requiring clinical reasoning, risk management, or multidisciplinary collaboration.

Such differentiation also depends on referral and shared care. Physicians and other healthcare professionals may identify medical risks, provide clinical guidance, and initiate referral, while appropriately qualified exercise professionals contribute exercise-specific assessment, prescription, progression, monitoring, and behavioral support (3, 11). Effective referral requires more than a formal route between services. It also depends on confidence in provider competence, clear role boundaries, information exchange, escalation procedures, organizational resources, and accountability for follow-up and participant safety (12). The organization of exercise services must therefore address both who provides each form of support and how responsibilities are coordinated across sectors.

Across Asia, governments and service organizations have incorporated physical activity and structured exercise for older adults into public health promotion, community services, fitness systems, disability-prevention initiatives, and long-term care programmes (1, 13). Their institutional location and intended purpose, however, vary considerably. Some programmes promote participation among generally healthy and independent older adults, whereas others aim to maintain function, prevent frailty or disability, support chronic disease management, or contribute to rehabilitation and care prevention. Exercise may consequently be led by fitness instructors, coaches, community programme personnel, preventive-care instructors, health exercise professionals, or personnel working alongside healthcare and long-term care providers. In this article, “exercise services for older adults” refers to structured and professionally supported exercise provision across these settings, with the required level of competence and supervision varying according to participant need and service purpose.

Research has established the benefits and design of physical activity and exercise interventions for older adults and has examined the roles, accreditation, and healthcare integration of exercise professionals (1, 2, 6, 7, 11). Comparative work has also identified substantial jurisdictional differences in professional recognition, scope of practice, and integration with healthcare. Zhou et al. (8), for example, compared exercise professions in Australia, mainland China, Taiwan, and Hong Kong, but addressed healthy populations, clinical populations, and athletes rather than differentiated services for older adults. Exercise referral research has similarly highlighted workforce skills, safety, accountability, organizational capacity, and local adaptation, without systematically comparing how aging Asian societies allocate responsibility across community promotion, disability prevention, long-term care, and clinically oriented exercise services (12).

The remaining gap concerns how institutional systems match heterogeneous older adult populations with appropriate workforce competencies and service pathways. It remains unclear when general fitness or community provision is considered sufficient, when more specialized exercise expertise is expected, and how referral or shared management with health and care professionals is organized. Ambiguity in these areas may lead either to insufficient support for older adults with complex needs or to unnecessary medicalisation that restricts access to community exercise. The resulting policy task is therefore to align participant need and service risk with provider preparation, permitted responsibilities, referral support, accountability, and interprofessional involvement.

To examine this challenge, the present study uses an integrative review and comparative institutional analysis of exercise-service workforce arrangements in Hong Kong, Taiwan, Japan, and Singapore. The cases represent contrasting combinations of general fitness, public health promotion, community care, long-term care prevention, and specialized health-exercise provision. The study addresses three research questions:

1. How do Hong Kong, Taiwan, Japan, and Singapore allocate professional roles for exercise services for older adults across different settings and levels of participant need?
2. To what extent are these services positioned within general fitness or community health promotion, long-term care or disability prevention, or specialized health-exercise practice involving assessment, risk management, referral, and interprofessional collaboration?
3. What competency domains, divisions of responsibility, referral arrangements, accountability mechanisms, and institutional conditions are needed to support safe, proportionate, and sustainable services for heterogeneous older adult populations?

By considering who provides different forms of exercise support, what competencies are expected, and how systems organize escalation and shared responsibility, this study conceptualizes older adult exercise as a differentiated workforce and service-delivery problem. It also develops a tiered model to inform professional preparation, policy design, and future empirical evaluation.

## 2 Literature review

### 2.1 Physical activity and structured exercise as strategies for healthy aging

Positioning physical activity at the center of healthy aging is important not merely because it can improve individual physiological indicators, but because it is directly related to whether older adults can maintain the functional ability required for everyday life. According to the World Health Organization, healthy aging is not simply the absence of disease, but the process of developing and maintaining the functional ability that enables wellbeing, including the ability to meet basic needs, remain mobile, make decisions, maintain relationships, and participate in society (1). The importance of physical activity therefore extends beyond disease prevention or fitness enhancement to delaying functional decline and supporting mobility, autonomy, and social participation in later life (1, 2).

Existing research has shown that regular physical activity is associated with a lower risk of functional limitation, slower progression of disability, and better performance in activities of daily living (14, 15). Fall prevention provides a particularly clear illustration of the relationship between structured exercise and functional ability. Systematic reviews indicate that exercise can reduce falls among community-dwelling older adults, especially when programmes provide a meaningful balance challenge and sufficient training dosage (48). Multicomponent programmes combining balance, resistance, functional, and mobility exercises may also improve lower-limb strength, postural control, and everyday physical performance (6, 16). From a healthy-aging perspective, exercise participation therefore contributes not only to greater activity levels, but also to safety, independence, confidence, and continued engagement in everyday life.

The aims and requirements of exercise provision, however, vary considerably across older adult populations. Relatively healthy, functionally independent, and medically stable older adults may benefit from general community fitness, recreational exercise, and health-promotion programmes. For these participants, the main objectives may be to increase activity, maintain fitness, promote social engagement, and prevent future decline. In contrast, older adults living with sarcopenia, frailty, impaired balance, fall risk, multimorbidity, cardiometabolic disease, functional deterioration, or recent medical treatment may require more individualized and progressively adjusted exercise interventions. In these circumstances, exercise may have treatment-related or rehabilitative aims, such as improving muscle strength and physical function, reducing fall risk, supporting disease management, restoring exercise tolerance, or delaying disability and care dependency (4, 6, 7).

These differences have direct implications for programme design and professional responsibility. Individualized exercise provision may require review of relevant health history, assessment of functional capacity and exercise tolerance, identification of contraindications, risk stratification, adaptation of exercise intensity and modality, symptom monitoring, and decisions concerning progression, temporary cessation, or referral. Clinical exercise physiology literature has shown that exercise professionals working with chronic or complex conditions may undertake exercise testing, functional assessment, individualized prescription, physiological monitoring, patient education, behavioral support, and communication with healthcare providers (7). The Australian model further demonstrates the importance of trained exercise professionals who can distinguish between low-risk activity support and exercise services requiring more advanced assessment, monitoring, and multidisciplinary management (10).

Exercise for older adults should therefore not be conceptualized as a single and uniform form of service. It ranges from general physical activity promotion and community-based exercise participation to structured programmes with preventive, functional, therapeutic, or rehabilitative objectives. The relevant workforce question consequently depends on the characteristics of the participants and the purpose and risk level of the service. The central issue is no longer simply whether older adults should be more physically active, but who is appropriately prepared to assess, prescribe, adapt, supervise, and monitor different forms of exercise provision, and when referral or shared management is required.

### 2.2 From exercise benefits to differentiated workforce arrangements

The question of who should provide exercise services in clinical and healthcare settings is not new. In the United States, clinical exercise physiologists have long been discussed as trained professionals who contribute to exercise testing, chronic disease management, rehabilitation, patient education, and supervised exercise delivery (7). In Australia, Accredited Exercise Physiologists have been formally recognized within the healthcare system and are educated to assess, prescribe, deliver, and evaluate exercise interventions for people with chronic conditions, injuries, disabilities, and complex health needs (8–10). In the United Kingdom, debates concerning exercise referral and the formal recognition of clinical exercise physiology have similarly highlighted the limitations of relying solely on general fitness qualifications for populations requiring clinical reasoning, individualized prescription, exercise testing, and behavior-change support (9).

This literature has also clarified that exercise provision should not be understood as the exclusive responsibility of either physicians or exercise professionals. Primary care physicians may be well positioned to assess general health concerns, initiate discussions about physical activity, provide medical guidance, and refer patients, but limited consultation time, knowledge, and referral resources may restrict their ability to provide detailed exercise prescription and long-term follow-up (3). Shared-care models therefore position appropriately qualified exercise professionals as complementary providers who can undertake assessment, individualized exercise prescription, progression, monitoring, and behavioral support, while maintaining bidirectional communication with physicians and referring participants back for medical review when necessary (11).

Exercise referral research has further shown that effective delivery depends on more than the formal existence of a referral pathway. Through the participatory development of an exercise referral scheme, Buckley et al. (12) identified existing service culture, workforce skills, safety, accountability, organizational resources, and local capacity as central to the translation of evidence into practice. These findings indicate that referral depends on mutual trust, timely information exchange, agreed escalation procedures, and clear accountability for participant safety. These questions have therefore received substantial attention in clinical exercise physiology, primary care, rehabilitation, and exercise referral research, particularly in Australia, the United Kingdom, and the United States.

Nevertheless, this broader international literature does not fully resolve the specific workforce questions surrounding exercise services for older adults in the selected Asian contexts. First, much of the clinical exercise literature focuses on chronic disease populations in general rather than on the heterogeneity of older adults across community health promotion, frailty prevention, long-term care prevention, rehabilitation support, and chronic disease management. Second, existing studies often examine a single occupation, service model, or national setting rather than comparing how different institutional systems allocate responsibility across general fitness, community care, public health, long-term care, and healthcare. Third, the presence of exercise-related qualifications does not necessarily clarify which professionals should serve relatively healthy older adults, which should manage participants with more complex functional or clinical needs, and how referral and shared management should operate between these levels.

Previous comparative research has begun to address these issues. Zhou et al. (8), for example, compared the roles and recognition of exercise professionals in Australia, mainland China, Taiwan, and Hong Kong, demonstrating substantial differences in accreditation, scope of practice, and integration into healthcare systems. However, that analysis examined exercise professionals serving healthy populations, clinical populations, and athletes and did not focus specifically on the differentiated service needs of older adults. Research examining instructors in community exercise settings has also shown that instructors act not only as demonstrators of movement, but as educators, leaders, facilitators of social relationships, and mediators of the participation experience (17). Older adults' continued participation may likewise be shaped by instructor characteristics, class atmosphere, peer interaction, and organizational support (18). These studies provide important insights into exercise delivery but do not fully explain how professional responsibilities should be distributed across different levels of participant need and risk.

The research gap should therefore not be framed as a general absence of attention to exercise workforce questions. Rather, it lies in the limited development and comparative analysis of differentiated workforce arrangements for exercise services for older adults in the selected Asian contexts. In Hong Kong, Taiwan, Japan, and Singapore, structured exercise may be located within community fitness, public health promotion, long-term care prevention, functional support, or specialized health exercise systems. Yet it remains unclear how these systems distinguish general activity facilitation from services requiring individualized assessment, risk management, monitoring, referral, or interprofessional collaboration.

A comparative institutional perspective is therefore needed to examine how professional roles are constructed across service settings, how workforce competencies are matched to participant needs and risks, and how responsibilities, service transitions, accountability, and institutional support are organized. Such an approach does not assume that a single profession should control all exercise services for older adults. Instead, it asks whether different systems provide proportionate and sufficiently clear arrangements for relatively healthy older adults as well as those with frailty, multimorbidity, functional limitations, or elevated clinical risk.

## 3 Methods

### 3.1 Study design and case selection

This study used an integrative review and comparative institutional analysis to examine professional roles, qualification pathways, competency expectations, referral arrangements, and governance structures for exercise services for older adults in Hong Kong, Taiwan, Japan, and Singapore. An integrative review was appropriate because the evidence extended beyond peer-reviewed research to policy documents, qualification frameworks, competency standards, programme materials, and official institutional sources.

The four high-income Asian jurisdictions were purposively selected according to three criteria: identifiable arrangements for delivering exercise or physical activity services to older adults; sufficient academic, policy, professional, and institutional documentation; and meaningful variation in how these services were positioned across fitness, public health, community care, long-term care prevention, and specialized health-exercise systems.

The cases represented contrasting institutional configurations. Hong Kong combined fitness certification with community-based aging initiatives; Taiwan linked fitness provision with health promotion and long-term care prevention; Japan included formal health-exercise qualifications and care-prevention arrangements; and Singapore relied mainly on state-supported public health, coaching, community, and skills-development systems. The cases were selected to support analytical comparison rather than regional representativeness.

### 3.2 Information sources and search date

The review drew on academic literature identified through PubMed, Scopus, Web of Science Core Collection, and Google Scholar, together with institutional and gray literature from relevant official, professional, educational, and programme-provider websites. All searches were conducted on 28 April 2026, using English, Traditional Chinese, Simplified Chinese, and Japanese terms as appropriate. The most recent authoritative institutional version available on that date was retained. Full search strategies are reported in Supplementary Table 1.

### 3.3 Academic database search strategies

Search strategies were adapted to the syntax and indexing structure of each database and covered five concept groups: (1) aging; (2) exercise or physical activity; (3) professional roles and workforce; (4) qualifications, competencies, referral, or governance; and (5) jurisdiction. Database-specific controlled vocabulary and title, abstract, keyword, or topic fields were used as appropriate.

For transparency and reproducibility, the complete PubMed search strategy was as follows:

((“Aged”[Mesh] OR (“older adult^*^” OR “older people” OR elderly OR senior^*^ OR “active aging” OR “active aging” OR “healthy aging” OR “healthy aging”)[Title/Abstract]) AND (exercise OR “physical activity” OR fitness OR “exercise prescription” OR “community exercise” OR “health exercise”)[Title/Abstract] AND (instructor^*^ OR coach^*^ OR trainer^*^ OR “exercise professional^*^” OR “fitness professional^*^” OR “exercise physiologist^*^” OR workforce)[Title/Abstract] AND (qualification^*^ OR certification OR credential^*^ OR competenc^*^ OR training OR “scope of practice” OR referral OR governance)[Title/Abstract] AND (“Hong Kong” OR Taiwan OR Japan OR Singapore)[Title/Abstract]).

The asterisk (^*^) was used as a truncation symbol to retrieve variant word endings, such as senior and seniors, coach and coaches, qualification and qualifications, and competence, competency, and competencies. Equivalent strategies were adapted to the relevant syntax and search fields of Scopus and Web of Science Core Collection. Shorter topic- and jurisdiction-specific combinations were used in Google Scholar to identify additional academic and institutional materials, which were verified against original or authoritative sources where possible. The complete database-specific search strings, Google Scholar search combinations, and jurisdiction-specific local-language and institutional website searches are provided in Supplementary Table 1.

### 3.4 Institutional and local-language searches

Institutional and gray-literature searches combined jurisdiction names with local-language terms relating to older adults, exercise, professional roles, qualifications, competency standards, health promotion, long-term care prevention, risk assessment, prescription, referral, and governance. Searches were conducted mainly in Traditional Chinese for Hong Kong and Taiwan, Japanese for Japan, and English for Singapore, with supplementary English searches across all jurisdictions. Official and authoritative sources providing verifiable information on qualifications, competencies, responsible institutions, service settings, scope of practice, referral, or governance were prioritized. Full search terms, local-language combinations, and principal website types are reported in Supplementary Table 1.

### 3.5 Eligibility criteria

Sources were included when they met the following criteria:

1. They concerned at least one of the four selected jurisdictions—Hong Kong, Taiwan, Japan, or Singapore—or provided directly relevant cross-jurisdictional evidence for interpreting exercise-service workforce arrangements.
2. They addressed exercise or structured physical activity services for older adults, including community health promotion, fitness instruction, long-term care or disability prevention, functional support, rehabilitation support, or chronic disease-related exercise provision.
3. They provided information on at least one relevant workforce or institutional dimension, including professional roles, qualifications, certification, competency standards, education or training pathways, institutional responsibility, service setting, scope of practice, role boundaries, risk assessment, exercise prescription or modification, monitoring, referral, or interprofessional collaboration.
4. They were peer-reviewed publications or were issued by government agencies, statutory or public bodies, recognized professional associations, qualification bodies, universities, official programme providers, or credible policy organizations.
5. Full text or sufficiently detailed and verifiable institutional information was available for structured data extraction.

No lower publication-date limit was imposed because historically important policy, qualification, and institutional documents were relevant to understanding the development of current workforce arrangements. All sources had to be published, updated, or publicly accessible by 28 April 2026.

Sources were excluded when they:

1. Reported only the effectiveness or outcomes of an exercise intervention without providing information on professional delivery, workforce roles, qualifications, or institutional arrangements;
2. Addressed general physical activity promotion without reference to professional, organizational, or institutional delivery;
3. Consisted of commercial advertising, informal blogs, unsupported opinion pieces, or news reports that could not be verified against an original or authoritative source;
4. Duplicated information available in a more current, complete, or authoritative source;
5. Provided insufficient information for structured data extraction; or
6. Were unrelated to older adults, exercise-service provision, workforce arrangements, or the selected jurisdictions and comparative aims of the study.

### 3.6 Screening and source selection

Screening followed a PRISMA-ScR-informed workflow adapted for an integrative review of academic and gray literature. After duplicate removal, titles, abstracts, executive summaries, webpage descriptions, or institutional summaries were screened against the eligibility criteria. Potentially eligible sources were then assessed in full.

Both authors independently completed initial screening and full-text assessment. Sources were retained for full-text review when either author considered them potentially eligible. Disagreements were resolved through discussion with reference to the eligibility criteria and complete source content; no third reviewer was required.

**Figure 1:**
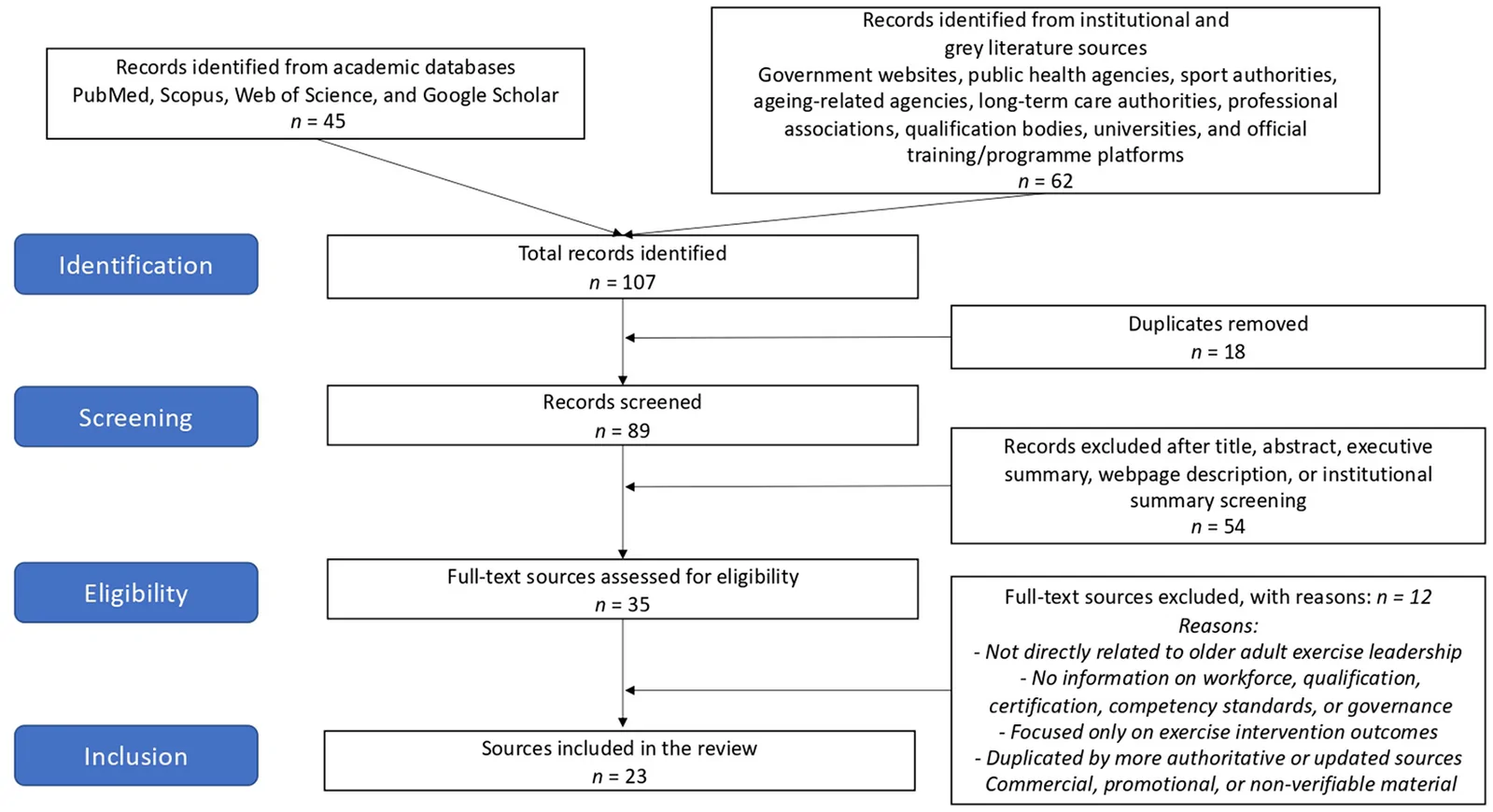
PRISMA-ScR-informed flow diagram for the identification and selection of academic, institutional, and gray-literature sources.

The searches identified 107 records, comprising 45 academic and 62 institutional or gray-literature records. After removing 18 duplicates, 89 records were screened and 54 were excluded. Thirty-five full-text sources were assessed, of which 12 were excluded for insufficient evidence on workforce or institutional arrangements. The final dataset comprised 23 sources: Hong Kong, *n* = 5; Taiwan, *n* = 6; Japan, *n* = 6; Singapore, *n* = 4; and cross-case sources, *n* = 2. The selection process is shown in Figure 1, and source characteristics are summarized in Table 1.

**Table 1:** Distribution and analytical characteristics of included sources by jurisdiction.

Jurisdiction/ Scope	*n*	Main document types	Main analytical relevance	References
Hong Kong	5	Qualification framework, programme documents, professional association documents	Fitness certification, older adult fitness ambassador training, senior functional training, community-based exercise leadership	(19–23)
Taiwan	6	National guideline, official training system, programme document, academic/policy sources	National fitness instructor system, preventive disability instructor training, senior fitness instructor training, community-based functional fitness	(24–29)
Japan	6	Qualification framework, national guideline, government policy report, professional association document	Health Exercise Instructor, care prevention exercise instructor, long-term care prevention, health promotion governance	(30–35)
Singapore	4	Official webpage, programme document, qualification framework, national guideline	CoachSG, Active Health, SkillsFuture training, national physical activity guidelines	(36–39)
Cross-case sources	2	Academic/policy sources	Regional aging policy and comparative physical activity context	(13, 42)
**Total**	**23**			

### 3.7 Source appraisal

Source characteristics and appraisal results are presented in Supplementary Table 2. Gray literature was assessed across the six AACODS domains of authority, accuracy, coverage, objectivity, date, and significance. Each domain was scored 0 or 1, with total scores of 5–6, 3–4, and 0–2 indicating higher, moderate, and lower confidence, respectively.

Academic publications were assessed across four study-specific domains informed by CASP principles: relevance, methodological clarity, credibility, and contribution to the comparative analysis. Each domain was scored 0 or 1, with scores of 4, 2–3, and 0–1 indicating higher, moderate, and lower confidence, respectively. These procedures were used to support transparent and structured judgement rather than as validated psychometric measures.

Both authors independently appraised all included sources, and disagreements were resolved through the process described in Section 3.6. Appraisal informed the interpretation and analytical weighting of the evidence rather than serving as a basis for automatic exclusion. Greater weight was given to sources that were authoritative, transparent, directly relevant, and sufficiently detailed to support the comparative analysis.

### 3.8 Data extraction and comparative institutional analysis

A standardized form was used to extract source characteristics, professional and organizational roles, target populations, service purposes and settings, qualification and training requirements, responsible institutions, scope of practice, risk management, referral and collaboration, and accountability arrangements. Both authors independently completed data extraction, and discrepancies were resolved using the process described in Section 3.6.

The analysis proceeded in four stages. First, within-case matrices were developed to map service systems, workforce roles, qualification pathways, responsible institutions, target populations, and service settings. Second, the extracted evidence was coded across four domains: (1) institutional location and service purpose; (2) professional and organizational responsibility; (3) qualification, training, and competency arrangements; and (4) risk management, referral, collaboration, and accountability.

Third, cross-case comparison examined how responsibilities were allocated across services for relatively independent older adults and those with greater functional or clinical complexity. Particular attention was given to whether systems distinguished general activity facilitation from services requiring individualized assessment, exercise prescription, monitoring, referral, or shared care.

Fourth, recurrent combinations of service location, workforce responsibility, qualification arrangements, and governance were synthesized into four overlapping analytical patterns: community-based fitness extension, prevention- or care-linked exercise provision, specialized health-exercise arrangements, and public-health-oriented coaching and programme delivery. These patterns were treated as analytical ideal types rather than mutually exclusive jurisdictional classifications; more than one pattern could therefore coexist within a jurisdiction.

Finally, competency domains were derived from recurring responsibilities identified across the 23 included sources and refined using international literature on clinical exercise physiology, exercise referral, and shared care. This literature provided interpretive context for framework development but was not included in the formal 23-source dataset.

## 4 Results

### 4.1 Cross-jurisdictional differences in target populations and service pathways

Table 2 compares the principal providers of exercise services for older adults, the populations served, relevant qualification and institutional pathways, and the extent to which risk stratification, referral, and interprofessional management were formally specified.

**Table 2:** Comparative evidence on target populations, workforce roles, and referral arrangements.

Jurisdiction	Main workforce	Primary target population	Main qualification or institutional pathway	Risk stratification and referral	Dominant model
Hong Kong	Fitness professionals, older adult fitness ambassadors, senior functional training coaches, and community programme personnel	Mainly community-dwelling older adults seeking fitness, functional training, or health promotion; some programmes address chronic disease-related exercise modification	Fitness certification, community ambassador training, university continuing education, and functional training certificates	Safety monitoring and basic assessment are included in some programmes, but no unified risk-stratification, referral, or shared-management pathway was identified	General fitness extension with community-based training
Taiwan	National fitness instructors, preventive and delayed disability instructors, senior fitness instructors, and community health promotion personnel	Community-dwelling older adults, including those at risk of frailty, disability, sarcopenia, or functional decline	National fitness certification, preventive disability training, vocational senior fitness training, and long-term care prevention programmes	Risk assessment and exercise modification are included in some pathways, but role boundaries, referral criteria, and interprofessional management remain fragmented	General fitness extension linked to long-term care prevention
Japan	Health Exercise Instructors, Health Exercise Practical Instructors, and care prevention exercise instructors	Older adults requiring health promotion, functional maintenance, or care prevention, including those with declining function or health-related risks	Health exercise qualifications, care prevention instructor training, and national health and long-term care policy	Strongest evidence of risk management and collaboration with health and medical professionals, although a uniform referral pathway for complex cases was not clearly documented	Specialized health-exercise and care-prevention model
Singapore	Coaches, Active Health Coaches, programme leaders, and senior fitness specialists	Mainly community-dwelling older adults receiving physical activity promotion, health coaching, or individualized exercise support	CoachSG, Active Health, SkillsFuture-supported training, and national physical activity guidelines	Some health assessment and individualized support are provided, but formal referral criteria and shared-care pathways for frailty or complex disease were not clearly specified	Public-health-promotion and coaching-development model

Across the four jurisdictions, service pathways were most clearly established for community-dwelling older adults who were generally independent or seeking health promotion, fitness improvement, and prevention of functional decline. All four systems included some aging-related training, safety guidance, functional assessment, or exercise modification. However, formal pathways were less consistently documented for older adults with frailty, chronic or multiple conditions, elevated fall risk, functional limitations, or other complex needs.

The principal cross-jurisdictional difference concerned the extent to which systems distinguished general exercise support from services requiring more advanced assessment, individualized prescription, monitoring, referral, or shared management. Hong Kong and Singapore relied mainly on fitness, coaching, community, and public health arrangements. Taiwan showed stronger links with disability prevention and long-term care policy, whereas Japan demonstrated the clearest connection among specialized exercise roles, care prevention, risk management, and health-professional collaboration. Nevertheless, none of the four systems provided a fully specified, system-wide pathway for allocating lower-risk and higher-risk older adults to different levels of exercise support.

### 4.2 Hong Kong: accessible fitness extension with limited clinical linkage

Hong Kong's older adult exercise workforce included fitness professionals, age-friendly fitness ambassadors, senior functional training coaches, university-trained instructors, and community programme personnel. Together, these pathways extended general fitness certification and community-based training into older adult services, creating multiple entry points for exercise leadership and participation (19–23).

Programme content ranged from fitness assessment, exercise instruction, functional training, and safety monitoring to aging physiology, exercise modification for chronic conditions, and exercise prescription. The depth, scope, and institutional authority of these competencies nevertheless varied across qualifications and programme-based courses. No unified framework was identified for distinguishing general community exercise provision from services intended for older adults with greater functional or clinical complexity.

Taken together, these arrangements reflect an accessible fitness-extension model with limited formal linkage to clinical services. The diversity of training and service pathways may support broad community access. However, the available documents did not clearly specify how participants should be assessed, what decisions each provider could make independently, or when exercise should be modified, suspended, or referred to another professional.

### 4.3 Taiwan: prevention-oriented expansion with fragmented referral arrangements

Taiwan's provision of exercise services for older adults drew on national fitness instructor certification, preventive and delayed disability training, vocational senior fitness programmes, and community health-promotion initiatives. The resulting service landscape extended beyond generally healthy older adults to include people at risk of frailty, sarcopenia, disability, or functional decline (24–29).

Within this landscape, the National Physical Fitness Instructor system provided a general certification base, while preventive and delayed disability programmes connected exercise delivery with public health and long-term care prevention. Senior fitness training added content on aging physiology, contraindications, functional assessment, progressive exercise design, and chronic-condition considerations. This combination gave greater prominence to frailty, functional vulnerability, and disability prevention than would be expected in a general fitness model alone.

The available documentation, however, did not set out an integrated pathway for stratifying older adults by functional or clinical risk, defining which providers could independently assess, prescribe, or substantially modify exercise, or determining when referral to medical, rehabilitation, or other health professionals should occur. This places Taiwan between general fitness provision and more specialized preventive care: its services extend further into functional risk, but responsibility for assessment, exercise modification, and onward referral remains divided across several systems.

### 4.4 Japan: the strongest documented linkage among exercise, care prevention, and health professionals

Among the four jurisdictions, Japan displayed the clearest documented institutional distinction between general physical activity promotion and health-related exercise or care-prevention services. Health Exercise Instructors, Health Exercise Practical Instructors, and care prevention exercise instructors operated within arrangements linking health promotion and functional maintenance with the prevention of care dependency and collaboration with health and medical professionals (30–35).

These qualification and training pathways encompassed individual assessment, safe programme design, muscle strengthening, balance improvement, cognitive and functional maintenance, risk management, and care prevention. Their content addressed declining function, care-prevention needs, and health-related risk more directly than programmes centered primarily on general community participation.

This comparatively specialized structure provided the clearest evidence of role definition and collaboration between the exercise and health sectors. Its professional and interprofessional linkages were nevertheless not equivalent to a fully integrated clinical exercise referral system. The available sources did not establish a consistent process for directing older adults with chronic, complex, or higher-risk conditions to appropriate exercise and healthcare services.

### 4.5 Singapore: coordinated public health delivery with unclear pathways for complex needs

Singapore integrated older adult exercise provision through national coaching infrastructure, Active Health services, SkillsFuture-supported training, and national physical activity guidance (36–39, 45). This nationally coordinated structure primarily served community-dwelling older adults through physical activity promotion, health coaching, programme delivery, and individualized exercise guidance.

Within this structure, Active Health Coaches provided health-related assessment and individualized support, while senior fitness training addressed aging physiology, exercise prescription, risk management, and considerations for special populations. These functions remained located mainly within coaching, public health, and continuing education rather than within a formally recognized clinical exercise profession.

The resulting model combined broad community reach with coordinated public health and coaching provision. Its service pathways were most clearly articulated for population-level promotion and guided community participation. By contrast, the available documentation provided less detail on how higher-risk participants were identified, when referral was required, and how exercise and healthcare providers coordinated care.

### 4.6 Cross-case synthesis

Three cross-case findings emerged. First, service arrangements were most clearly documented for community-dwelling older adults receiving fitness, health-promotion, functional-maintenance, or preventive support. Second, all four jurisdictions incorporated some aging-specific content into fitness, coaching, or care-prevention training, although the depth and institutional authority of this preparation varied. Third, the distinction between general exercise provision and services requiring advanced assessment, monitoring, referral, or interprofessional involvement remained inconsistently defined.

The jurisdictions differed in how these services were organized. Hong Kong relied mainly on fitness and community pathways, with limited documented links to formal risk assessment and referral. Taiwan extended provision into frailty and disability prevention, but responsibilities remained distributed across sport, health, long-term care, and vocational systems. Japan demonstrated the strongest documented specialization and interprofessional linkage, whereas Singapore showed the clearest national coordination of public health and coaching provision. Across all four cases, arrangements for participation and prevention were more clearly documented than those for older adults with complex needs. The principal gap was the lack of clear guidance on who should assess risk, what decisions different providers could make, when another service should become involved, and who remained responsible for follow-up.

## 5 Discussion

These findings indicate that exercise services for older adults cannot be treated as uniform. The level of support required depends on participants' health status, functional capacity, and service needs. Relatively healthy and independent individuals may be appropriately supported through accessible community-based exercise, whereas those with frailty, recurrent falls, multimorbidity, or complex functional limitations may require individualized assessment, exercise modification, closer monitoring, referral, or interprofessional management (4, 6, 7).

Across the four jurisdictions, arrangements were clearer for population-level promotion and prevention than for participants requiring more individualized or clinically informed support. The main difference lay in how explicitly each system connected participant complexity with the expertise and support available. Japan showed the strongest documented connection between specialized exercise roles and health-professional involvement. In the other jurisdictions, responsibility remained more dispersed across fitness, community, coaching, public health, and long-term care settings. None provided a consistently documented process for allocating higher-risk participants to an appropriate level of support.

### 5.1 Community exercise promotion and clinically oriented exercise services

Community exercise promotion and clinically oriented exercise services are complementary but require different levels of expertise. Programmes for relatively healthy and independent older adults may appropriately focus on increasing physical activity, maintaining mobility and basic fitness, supporting social participation, and preventing functional decline. Fitness instructors, coaches, trained community personnel, ambassadors, and peer leaders may contribute to these aims when they possess aging-related knowledge, communication and safety skills, and the ability to recognize when referral is needed (17, 18, 40, 41). By contrast, older adults with frailty, multimorbidity, recurrent falls, unstable chronic conditions, or substantial functional limitations may require individualized prescription, closer monitoring, condition-specific modification, and communication with healthcare or rehabilitation professionals (7, 9, 11).

The Australian model illustrates how this distinction can be institutionalized. General exercise and fitness services are differentiated from those delivered by Accredited Exercise Physiologists, who are prepared to provide exercise interventions for people with chronic disease, injury, disability, or complex health needs. This arrangement supports shared care in which medical professionals identify relevant clinical risks, while appropriately qualified exercise professionals provide exercise-specific assessment, prescription, progression, monitoring, and behavioral support (8, 10, 11). The model does not imply that all older adults require clinical exercise services; rather, it demonstrates how professional responsibilities may be differentiated according to participant need.

The four jurisdictions varied in how clearly this distinction was reflected in their institutional arrangements. Hong Kong and Singapore provided accessible fitness, community, coaching, and public health pathways, but referral arrangements for participants with complex needs were less clearly documented. Taiwan more explicitly incorporated frailty, disability prevention, and functional decline, although responsibilities remained distributed across several sectors. Japan showed the strongest documented linkage among exercise provision, care prevention, risk management, and health-professional collaboration. These differences suggest that the central issue is not whether one workforce model is universally superior, but whether each system matches provider competence and service intensity to participant need. Insufficient differentiation may expose higher-risk older adults to inadequate support, whereas excessive medicalisation may restrict accessible community participation among lower-risk individuals (11, 12, 40).

### 5.2 Risk stratification, referral, and shared care

The findings revealed uncertainty at four decision points: identifying participant risk, determining what each provider could manage independently, deciding when another service should become involved, and maintaining continuity across sectors. Aging-related training alone does not constitute formal risk stratification. Although programmes may address chronic conditions, falls, and exercise safety, they do not necessarily specify how participants should be classified according to medical stability, functional capacity, frailty, fall risk, or need for professional supervision (3, 7, 10). Similarly, certification alone does not clarify which providers may independently assess function, prescribe or substantially modify exercise, manage multiple conditions, or continue services when warning signs emerge. Clearer scope-of-practice boundaries are therefore needed to connect provider preparation with permitted responsibilities (8).

Referral should be understood as a coordinated process rather than a one-directional transfer after a problem occurs. Effective pathways should specify eligibility, assessment, safety procedures, referral thresholds and destinations, professional accountability, communication, follow-up, and continuity across services. Their implementation also depends on multidisciplinary involvement, clearly defined responsibilities, adequate resources, and integration with existing service structures (12). Within such pathways, healthcare professionals may identify medical risks and clinical priorities, while appropriately qualified exercise professionals provide exercise-specific assessment, prescription, supervision, behavioral support, and feedback to the healthcare team (7, 11).

Involving multiple sectors is not itself problematic and may improve access and service capacity. Difficulties arise when systems do not specify who assesses risk, who provides each level of exercise support, when referral or escalation is required, how information is shared, and who is accountable for monitoring and follow-up. Interprofessional education and collaboration may help clarify these responsibilities across exercise, rehabilitation, healthcare, long-term care, and community services (13, 42, 43).

### 5.3 Workforce models and programme outcomes: a future research agenda

Future research should examine whether, and under what conditions, different workforce models are associated with different programme outcomes. Rather than assuming that occupational title or formal qualification alone determines service quality, studies should assess how provider competence interacts with participant health and functional risk, exercise content and dose, service setting, organizational resources, and access to referral or clinical support (7, 9, 12). Provider competencies in assessment, exercise modification, monitoring, motivation, and interprofessional communication should be measured directly rather than inferred from professional title.

Prospective comparative and implementation studies should evaluate programmes delivered by community personnel, general fitness providers, specialized exercise professionals, and interprofessional teams. Outcomes should include physical function, falls and adverse events, safety, adherence, psychosocial wellbeing, accessibility, cost, referral completion, and continuity of care (6, 11, 18, 41). Particular attention should be given to whether different workforce arrangements are appropriate for different participant groups, including relatively healthy older adults and those with frailty, multimorbidity, recurrent falls, or clinically significant functional limitations.

### 5.4 A tiered competency and service framework

The findings do not suggest that every jurisdiction should establish a single new occupation or additional certificate for older adult exercise. A more feasible approach is to align participant risk and service needs with provider competence, scope of practice, referral arrangements, and accountability. The proposed tiered model was derived from the cross-jurisdictional findings and informed by literature on clinical exercise physiology, exercise referral, risk-matched provision, and shared care. It is intended as a conceptual guide rather than an empirically validated classification system (7, 10, 11).

At the first level, trained community personnel, ambassadors, and general fitness or coaching staff may support relatively healthy and functionally independent older adults through physical activity promotion and basic exercise instruction. Required competencies include aging-related knowledge, inclusive communication, basic exercise safety, recognition of warning signs, and referral literacy. Their primary role is to facilitate safe participation, appropriate progression, social engagement, and sustained involvement rather than clinical assessment or management.

At the second level, providers working with older adults who have stable chronic conditions, early frailty, sarcopenia, functional decline, or increased fall risk require more advanced preparation. Relevant competencies include functional assessment, individualized exercise prescription, programme modification and progression, symptom and safety monitoring, documentation, and communication with health or care professionals. Providers should also recognize when participants require expertise beyond their own preparation and know how to direct them to appropriate services.

At the third level, older adults with unstable, complex, or clinically significant conditions may require services delivered or jointly managed by professionals with advanced clinical exercise, rehabilitation, or health qualifications. Formal communication and joint management with physicians, allied health professionals, long-term care providers, and community services are particularly important at this level (7, 9, 11).

Across all three levels, the relevant competency domains include assessment, exercise design and modification, monitoring and safety management, motivation and sustained participation, referral and interprofessional collaboration, and digital or remote support (44, 47). However, the required depth of these competencies should vary according to participant risk, service purpose, and professional responsibility. The framework is intended to avoid both treating all older adult exercise as a low-risk community activity and assuming that all older adults require medically oriented services. Its central principle is to match the intensity of assessment, exercise support, monitoring, and collaboration to the participant's health and functional profile.

## 6 Implications for research, practice, and policy

The principal implication is that exercise services should be matched to older adults' health and functional needs rather than organized according to age alone. Research, practice, and policy should therefore ensure that the intensity of assessment, supervision, and professional support is proportionate to participant complexity.

### 6.1 Implications for research

Future research should examine how participant risk, provider competence, service setting, and referral support jointly influence programme outcomes. Studies should clearly report participants' health and functional profiles and compare services delivered by community personnel, general fitness providers, specialized exercise professionals, and interprofessional teams. Outcomes should extend beyond physical and functional measures to include safety, adverse events, adherence, psychosocial wellbeing, accessibility, cost, referral completion, and continuity of care. Comparisons should also account for exercise content, dose, participant characteristics, and clinical support to avoid attributing outcomes to occupational title alone.

The proposed tiered model requires empirical validation across jurisdictions. Expert consensus, curriculum analysis, stakeholder consultation, and implementation studies could assess the appropriateness of its service levels and competency domains. Particular attention should be given to defining practical referral thresholds and clarifying which assessments, exercise modifications, and monitoring responsibilities are appropriate at each workforce level.

### 6.2 Implications for practice

Exercise providers should determine the level of support required before assigning older adults to a programme. Community and fitness organizations should use basic pre-participation screening to identify relevant health concerns, functional limitations, fall risk, and warning signs (3, 7, 10). Such screening is not intended to provide a clinical diagnosis, but to determine whether community exercise is appropriate or whether further assessment or referral is needed.

For relatively healthy and independent older adults, programmes may emphasize safe participation, appropriate progression, motivation, social support, and sustained engagement (17, 18, 40, 41). Providers should possess basic competencies in aging, communication, exercise safety, recognition of adverse signs, and referral. Older adults with stable chronic conditions, early frailty, sarcopenia, functional decline, or increased fall risk may require more individualized assessment, prescription, modification, progression, and monitoring, supported by appropriate documentation and communication with health or care professionals (4, 6, 7).

Participants with unstable symptoms, complex multimorbidity, recurrent falls, substantial functional impairment, or other clinically significant conditions may require exercise services delivered or jointly managed by personnel with advanced clinical exercise, rehabilitation, or health qualifications (7, 9, 11). Exercise professionals should complement rather than replace physicians, rehabilitation practitioners, nurses, and other healthcare providers. Their exercise-specific expertise should be integrated into clear referral, communication, monitoring, and shared-care arrangements to support safe adaptation and continuity of care (9, 11, 42).

### 6.3 Implications for policy

Policy should move beyond promoting physical activity and establish how exercise services will be organized for older adults with different health and functional needs. Because a single qualification is unlikely to cover the full continuum of service complexity, a tiered policy framework may be more appropriate (8–10). Community-level policy should support accessible programmes delivered by personnel with basic aging-specific and safety competencies. Intermediate pathways should prepare providers to deliver individualized exercise for older adults with stable chronic conditions or emerging functional risks, while clinical-level policy should clarify the roles of specialized exercise professionals and their relationships with healthcare, rehabilitation, and long-term care services.

Across these levels, policy makers and professional bodies should define minimum competencies, scope-of-practice boundaries, risk-identification criteria, referral thresholds and destinations, communication and documentation procedures, adverse-event responsibilities, continuing professional development, quality assurance, and accountability arrangements. These provisions are necessary to ensure that providers undertake only those assessments, exercise decisions, and monitoring responsibilities appropriate to their preparation.

Cross-sector coordination is particularly important because exercise services for older adults span sport, public health, community care, rehabilitation, healthcare, and long-term care prevention (11, 12, 42). Shared standards and referral mechanisms could preserve accessible community provision for lower-risk older adults while enabling those with more complex needs to receive specialized exercise or interprofessional support. The policy priority is to expand service capacity while ensuring that higher-risk participants can access more specialized and coordinated support.

## 7 Limitations

Several limitations should be considered. First, the four jurisdictions were purposively selected to provide institutional contrast rather than regional representativeness. All are relatively high-income and well-resourced Asian settings with comparatively formalized service systems. The findings should therefore not be generalized to Asia as a whole, particularly to lower-resource settings or contexts that rely more heavily on private, community-based, family-based, or informal exercise provision.

Second, this document-based integrative review was limited to publicly available academic, policy, professional, qualification, training, and programme materials. The availability, detail, language, and institutional transparency of these sources varied across jurisdictions, and some local concepts lacked direct English equivalents. Although local-language searches and authoritative sources were used, relevant documents and informal practices may have been missed. The review also could not determine how documented policies, qualifications, divisions of responsibility, referral procedures, insurance arrangements, and cross-sector coordination were implemented in practice. The absence of documentary evidence should therefore not be interpreted as evidence that such arrangements do not exist.

Third, the workforce patterns and proposed tiered model are interpretive constructs derived from the reviewed evidence and related international literature. They represent overlapping analytical patterns rather than fixed jurisdictional classifications or validated indicators of professional practice. The review did not directly assess practitioner competence, service quality, programme fidelity, participant experience, or whether providers with different qualifications produce different physical, functional, safety, behavioral, psychosocial, or service outcomes. The proposed alignment among participant risk, provider competence, and service level therefore remains conceptual and requires empirical validation.

Finally, the analysis focused primarily on formal institutional and workforce arrangements and did not systematically examine the broader cultural, social, and organizational factors that may shape service implementation. Attitudes toward aging, professional authority, family and community responsibility, preventive care, healthcare referral, and interprofessional collaboration may influence how exercise roles are recognized and how older adults engage with services (46). Future comparative and implementation research should therefore examine these contextual influences and test the applicability of the proposed framework in more diverse settings, including lower-resource, highly populated, and socioeconomically varied Asian contexts.

## 8 Conclusion

This study compared workforce arrangements for older adult exercise services in Hong Kong, Taiwan, Japan, and Singapore. Across the four jurisdictions, services were organized through different combinations of fitness, community, public health, coaching, care-prevention, and health-related systems. Pathways for relatively healthy and independent older adults were generally clearer than those for people with frailty, multimorbidity, recurrent falls, functional decline, or other complex health needs. Japan demonstrated the strongest documented links among specialized exercise roles, care prevention, risk management, and health-professional collaboration, although none of the jurisdictions provided a consistently documented process for directing higher-risk older adults to appropriate exercise and healthcare support. These findings suggest that the central issue is not simply who leads exercise, but whether the level of assessment, supervision, and professional support is proportionate to each participant's needs. Relatively healthy older adults may be appropriately supported through accessible community programmes, whereas those with greater functional or clinical complexity may require individualized assessment, exercise modification, closer monitoring, specialized expertise, or interprofessional management. The proposed tiered model offers a basis for differentiating community-level support, advanced exercise provision, and clinically coordinated care, but it requires empirical validation. Future research should examine how different workforce arrangements influence safety, physical function, participation, accessibility, and continuity of care. Policy and practice should also clarify responsibilities for participant assessment, exercise modification, escalation, and follow-up so that older adults receive an appropriate level of support from suitably prepared personnel.

## Data Availability

The original contributions presented in the study are included in the article/Supplementary material, further inquiries can be directed to the corresponding author.
