# Supplementary material for: Who leads exercise for older adults? A comparative policy and workforce analysis of older adult exercise leadership in Hong Kong, Taiwan, Japan, and Singapore
Source: Front Public Health. 2026 Aug 7;14:1879234. doi: 10.3389/fpubh.2026.1879234 (PMC13493249; doi:10.3389/fpubh.2026.1879234)
Supplement: Supplementary file 1 [file Data_Sheet_1.pdf]

Supplementary Table 1. Complete Academic Database, Institutional Website, and Local-Language Search Strategies

Search date: 28 April 2026

Information source / jurisdiction	Search field or website type	Language	Exact search string or principal search combinations
PubMed	MeSH and Title/Abstract	English	(("Aged"[Mesh] OR "older adult*"[Title/Abstract] OR "older people"[Title/Abstract] OR elderly[Title/Abstract] OR senior*[Title/Abstract] OR "active ageing"[Title/Abstract] OR "active aging"[Title/Abstract] OR "healthy ageing"[Title/Abstract] OR "healthy aging"[Title/Abstract]) AND (exercise[Title/Abstract] OR "physical activity"[Title/Abstract] OR fitness[Title/Abstract] OR "exercise prescription"[Title/Abstract] OR "community exercise"[Title/Abstract] OR "health exercise"[Title/Abstract]) AND (instructor*[Title/Abstract] OR coach*[Title/Abstract] OR trainer*[Title/Abstract] OR "exercise professional*"[Title/Abstract] OR "fitness professional*"[Title/Abstract] OR "exercise physiologist*"[Title/Abstract] OR workforce[Title/Abstract]) AND (qualification*[Title/Abstract] OR certification[Title/Abstract] OR credential*[Title/Abstract] OR competenc*[Title/Abstract] OR training[Title/Abstract] OR "scope of practice"[Title/Abstract] OR referral[Title/Abstract] OR governance[Title/Abstract]) AND ("Hong Kong"[Title/Abstract] OR Taiwan[Title/Abstract] OR Japan[Title/Abstract] OR Singapore[Title/Abstract]))
Scopus	TITLE-ABS-KEY	English	TITLE-ABS-KEY(("older adult*" OR "older people" OR elderly OR senior* OR "active ageing" OR "active aging" OR "healthy ageing" OR "healthy aging") AND (exercise OR "physical activity" OR fitness OR "exercise prescription" OR "community exercise" OR "health exercise") AND (instructor* OR coach* OR trainer* OR "exercise professional*" OR "fitness professional*" OR "exercise physiologist*" OR workforce) AND (qualification* OR certification OR credential* OR competenc* OR training OR "scope of practice" OR referral OR governance) AND ("Hong Kong" OR Taiwan OR Japan OR Singapore))
Web of Science Core Collection	Topic (TS)	English	TS=(("older adult*" OR "older people" OR elderly OR senior* OR "active ageing" OR "active aging" OR "healthy ageing" OR "healthy aging") AND (exercise OR "physical activity" OR fitness OR "exercise prescription" OR "community exercise" OR "health exercise") AND (instructor* OR coach* OR trainer* OR "exercise professional*" OR "fitness professional*" OR "exercise physiologist*" OR workforce) AND (qualification* OR certification OR credential* OR competenc* OR training OR "scope of practice" OR referral OR governance) AND ("Hong Kong" OR Taiwan OR Japan OR Singapore))
Google Scholar	Relevance-ranked search results	English	“older adults” exercise instructor qualification “Hong Kong”<br>“older adults” exercise workforce certification Taiwan<br>“older adults” “health exercise instructor” Japan

Information source / jurisdiction	Search field or website type	Language	Exact search string or principal search combinations
			"older adults" exercise coach qualification Singapore<br>"older adults" exercise referral professional role<br>"clinical exercise physiology" older adults workforce<br>"exercise professional" older adults scope of practice<br>"older adults" exercise competency framework
Hong Kong institutional and professional websites	Government, sport, ageing, university, professional-association, and programme websites	English; Traditional Chinese	Principal English and Traditional Chinese combinations included:<br>("Hong Kong" OR 香港) AND ("older adults" OR elderly OR seniors OR 長者 OR 高齡者 OR 銀髮族) AND (exercise OR fitness OR physical activity OR 運動 OR 體適能 OR 功能性體適能) AND (instructor OR coach OR trainer OR qualification OR certification OR competency OR 運動指導員 OR 體適能指導員 OR 教練 OR 資格 OR 證照 OR 認證 OR 職能基準).<br>Additional combinations: 高齡運動指導; 銀髮族體適能指導; 長者運動課程; 社區長者運動; 運動處方; 風險評估; 轉介.
Taiwan institutional and professional websites	Government, sport, health, long-term-care, university, and training websites	Traditional Chinese	Principal Traditional Chinese combinations included:<br>(高齡者 OR 長者 OR 銀髮族 OR 樂齡) AND (運動 OR 體適能 OR 健康促進 OR 功能性體適能) AND (運動指導員 OR 體適能指導員 OR 教練 OR 高齡運動指導 OR 銀髮族體適能指導) AND (資格 OR 證照 OR 認證 OR 職能基準 OR 訓練課程 OR 運動處方 OR 風險評估 OR 轉介).<br>Additional combinations: 長期照護; 預防及延緩失能; 社區照護; 高齡者健康促進; 高齡體適能指導員.
Japan institutional and professional websites	Government, health-promotion, care-prevention, professional-association, and training websites	Japanese; English	Principal Japanese combinations included:<br>(高齢者) AND (運動 OR 身体活動 OR 健康づくり) AND (運動指導 OR 健康運動指導士 OR 介護予防運動指導員) AND (資格 OR 養成講座 OR 運動処方 OR 介護予防 OR 地域健康づくり).<br>Additional combinations: 高齢者 運動 指導 資格; 健康運動指導士 高齢者; 介護予防 運動 指導員; 高齢者 運動処方; 地域 高齢者 健康づくり.

Information source / jurisdiction	Search field or website type	Language	Exact search string or principal search combinations
Singapore institutional and professional websites	Government, Sport Singapore, Health Promotion Board, SkillsFuture, coaching, and programme websites	English	Principal English combinations included:<br>Singapore AND ("older adults" OR seniors OR ageing OR aging) AND (exercise OR physical activity OR fitness OR community exercise OR active health) AND (coach OR instructor OR trainer OR qualification OR certification OR competency OR SkillsFuture OR CoachSG OR referral OR governance).<br>Additional combinations: seniors exercise programme Singapore; older adults fitness instructor Singapore; Active Health older adults; CoachSG senior fitness; SkillsFuture exercise older adults.
