# Supplementary material for: Who leads exercise for older adults? A comparative policy and workforce analysis of older adult exercise leadership in Hong Kong, Taiwan, Japan, and Singapore
Source: Front Public Health. 2026 Aug 7;14:1879234. doi: 10.3389/fpubh.2026.1879234 (PMC13493249; doi:10.3389/fpubh.2026.1879234)
Supplement: Supplementary file 2 [file Data_Sheet_2.pdf]

Supplementary Table 2. Document Audit Trail and Quality Appraisal of Included Sources

Jurisdiction	Source	Appraisal framework	Appraisal score	Confidence level	Rationale
Hong Kong	Physical Fitness Association of Hong Kong, 2026	AACODS	5/6	High	Professional association qualification framework; highly relevant to fitness certification
Hong Kong	Jockey Club “Age-friendly Fitness” Programme, 2025	AACODS	4/6	Moderate	Programme document related to older adult fitness, but lower institutional authority than government sources
Hong Kong	Hong Kong Baptist University SCE, 2025	AACODS	5/6	High	University continuing education course with credible training information
Hong Kong	Social Innovation and Entrepreneurship Development Fund, 2024	AACODS	4/6	Moderate	Social innovation / programme-based source; useful for community model, but not a formal qualification system
Hong Kong	Hong Kong Sport and Psychology Association, 2023	AACODS	4/6	Moderate	Professional association course; useful, but certification authority should be interpreted cautiously
Taiwan	Sports Administration, Ministry of Education, 2025	AACODS	6/6	High	Official national fitness instructor system; core evidence

Taiwan	Health Promotion Administration, MOHW, 2025	AACODS	6/6	High	Official preventive and delayed disability instructor training system; core evidence
Taiwan	Workforce Development Agency, 2024	AACODS	5/6	High	Official vocational training source related to senior fitness training
Taiwan	Hualien County Health Bureau, 2021	AACODS	4/6	Moderate	Local health bureau source; policy-related but more limited in representativeness
Taiwan	Ministry of Science and Technology, 2016	AACODS	4/6	Moderate	Policy / research project source; useful as supplementary evidence, but not a direct qualification system
Taiwan	National Taiwan Normal University, 2023	AACODS	4/6	Moderate	University-hosted research or programme document; useful for community functional fitness evidence.
Japan	Japan Health Promotion and Fitness Foundation, 2026	AACODS	6/6	High	Health Exercise Instructor qualification system; core evidence
Japan	Tokyo Metropolitan Institute for Geriatrics and Gerontology, 2026	AACODS	6/6	High	Care prevention exercise instructor training; core evidence

Japan	Japan Care Prevention Association, 2024	AACODS	5/6	High	Professional association source highly relevant to care prevention
Japan	Ministry of Health, Labour and Welfare, 2006	AACODS	5/6	High	Official historical institutional document; older but institutionally significant
Japan	Ministry of Health, Labour and Welfare, 2023	AACODS	6/6	High	Recent official physical activity guideline; core policy evidence
Japan	Global Observatory of Long-Term Care, 2023	AACODS	5/6	High	Long-term care system policy source; useful for institutional background
Singapore	Sport Singapore, 2025	AACODS	6/6	High	Official CoachSG source; core evidence
Singapore	Sport Singapore, 2026	AACODS	6/6	High	Official Active Health source; core evidence
Singapore	SkillsFuture Singapore, 2025	AACODS	5/6	High	Official skills-training platform; relevant to training pathways
Singapore	Health Promotion Board, 2022	AACODS	6/6	High	National physical activity guideline; core policy evidence
Cross-case	Health Resources and Services Administration, 2019	AACODS	4/6	Moderate	Useful for interprofessional or care workforce background, but not a core Asian case source

Cross-case	O’Donnell et al., 2025	CASP-informed	4/4	High	Peer-reviewed academic article providing relevant evidence on interprofessional practice and role clarity.
